# Direct Visualization of the Hydration Layer on Alumina Nanoparticles with the Fluid Cell STEM *in situ*

**DOI:** 10.1038/srep09830

**Published:** 2015-05-21

**Authors:** Emre Firlar, Simge Çınar, Sanjay Kashyap, Mufit Akinc, Tanya Prozorov

**Affiliations:** 1 Division of Materials Science and Engineering, US DOE Ames Laboratory, Ames, IA, 50011, USA; 2 Department of Materials Science and Engineering, Iowa State University, Ames, IA, 50011, USA

## Abstract

Rheological behavior of aqueous suspensions containing nanometer-sized powders is of relevance to many branches of industry. Unusually high viscosities observed for suspensions of nanoparticles compared to those of micron size powders cannot be explained by current viscosity models. Formation of so-called hydration layer on alumina nanoparticles in water was hypothesized, but never observed experimentally. We report here on the direct visualization of aqueous suspensions of alumina with the fluid cell *in situ.* We observe the hydration layer formed over the particle aggregates and show that such hydrated aggregates constitute new particle assemblies and affect the flow behavior of the suspensions. We discuss how these hydrated nanoclusters alter the effective solid content and the viscosity of nanostructured suspensions. Our findings elucidate the source of high viscosity observed for nanoparticle suspensions and are of direct relevance to many industrial sectors including materials, food, cosmetics, pharmaceutical among others employing colloidal slurries with nanometer-scale particles.

The nanometer-sized oxide particles represent a class of materials with important applications in numerous fields of industry ranging from catalysis^1^, nanofluids^2^, advanced ceramics^3^, paints^4^, mining and soil^5^, gas sensors^6^, UV absorption materials^7^, cement^8^, food^9^ and cosmetics^10^, and numerous others^11^,^12^,^13^,^14^,^15^. Nanostructured alumina is one of the most commonly used, extensively studied ceramic oxides. Water has been frequently used as a solvent for fine oxide suspensions due to its safety, low cost and waste disposal properties compared to those of organic solvents^16^.

It was long proposed, that in aqueous suspensions and slurries containing nanometer-sized particles, water can be present in two forms: free water in solution and water bound to the particle surface^17^,^18^,^19^. The viscous behavior of the system could be attributed to water–particle and particle–particle interactions at the nanometer scale. Brownian, Coulombic and hydrodynamic forces are presumed to be the main forces affecting the rheological behavior of colloidal suspensions of charged particles. The unexpectedly high viscosities observed for these slurries were attributed to water−particle interactions at the nanometer scale. Existing rheological models do not accurately predict the viscosities of nanoparticle suspensions^20^,^21^. In fact, most models would not even incorporate particle size, necessitating a need in establishing a better understanding of behavior of nanoparticles suspended in aqueous media. Since neither electrostatic, steric, nor electrosteric stabilization mechanisms are able to explain the observed high viscosities and subsequent viscosity reductions with non-ionic additives, the “bound layer” (a. k. a “hydrated layer”) model, has been offered. This model was supported by several indirect measurements, in particular low temperature differential scanning calorimetry (LT-DSC)^17^,^22^,^23^,^24^ and colloidal probe atomic force microscopy (CP-AFM)^25^. However, even though the existence of hydrated layer has been hypothesized previously, it has never been visualized directly, largely due to the limitations posed by the common analytical techniques. A direct observation of hydrated layer (HL) formed around suspended nanoparticles is sorely needed to describe the viscosity of nanoparticle suspensions quantitatively.

Transmission Electron Microscopy (TEM) is a powerful tool uniquely suitable for structural characterization of a variety of nanometer-sized structures with high spatial resolution. However, it traditionally does not allow imaging in native liquid or atmospheric environments because of the high vacuum requirements of the instrument. TEM specimens are routinely prepared by placing a droplet of nanoparticle suspension on a suitable electron microscopy (EM) grid. The TEM examination of specimens prepared in this manner yields important information about the particle size and structure, however, evaporation of solvent can induce undesired aggregation of suspended nanoparticles, potentially leading to distorted view of state of the particles in liquid. Use of cryogenic TEM (cryo-TEM) analysis, where the specimens are plunged into liquefied ethane and visualized in a vitrified state, provides an alternative method for characterization of aqueous particles suspensions. This technique allows imaging of specimens retaining the original structural arrangement of its components; however, it is restricted to imaging in a literally frozen mode and cannot provide information about the dynamic processes taking place in liquid^26^,^27^. To understand the hydration behavior of nanosized particles, the system must be characterized in liquid.

Using the fluid cell holder Scanning Transmission Electron Microscopy (STEM) platform, it is possible to image the nanoscale suspensions in liquid with high resolution, ensuring the specimen remains in its natural, fully hydrated state, free of artifacts associated with the conventional sample preparation. Such an observation cannot be carried out by *any* *other technique*. The experimental setup typically consists of a microfluidic chamber comprised of two silicon chips with electron-transparent silicon nitride (Si_3_N_4_) windows contained in a hermetically sealed TEM holder. A thin liquid layer containing the specimen is sealed and maintained between the Si_3_N_4_ windows. When working with the continuous flow fluid cell, the imaging can be carried out under conditions most closely resembling those in colloidal suspensions. Moreover, use of High Angle Annular Dark Field (HAADF) imaging mode provides sufficiently high imaging contrast between water and suspended inorganic component, and allows direct *in situ* visualization of the individual nanoparticles in liquid.

In this work, nanometer-sized alumina powder was employed as a model system. Alumina nanopowder is comprised of polydisperse spherical particles and is free from bulk impurities. HAADF-STEM imaging was employed to obtain compositional and morphological information of the analyzed sample, given that the intensity of the HAADF-STEM images depends primarily on the atomic number (Z) and thickness of the specimen. Combined with the Electron Energy Loss Spectroscopy (EELS), HAADF-STEM imaging ensured comprehensive microstructural and localized chemical analyses of suspended nanoparticles both *in situ* and *ex situ*. We observed the hydration layer formed over the surface of aggregates in aqueous alumina suspensions and examined the effect of this phenomenon on rheological behavior of the system. The significant increase in the viscosity upon incorporation of nanoparticles is compared to the values predicted by the Krieger-Dougherty equation^28^, based on the volume fraction of the nanoparticles, the maximum packing fraction of the particles in the slurry, and the intrinsic viscosity of these particles. We have determined that the conventional models underestimate the observed viscosities. The present study showed unequivocally that in aqueous solutions alumina nanoparticles aggregate to form clusters with a hydrated layer and varying aspect ratio that are significantly larger than the primary particles. These clusters with the surrounding hydration cloud should be considered as new particles resisting to the flow under applied shear stress. These clusters increase the effective solids content of the suspensions, effectively decreasing the available free liquid carrier, hence leading to exceptionally high suspension viscosities.

## Results

**Figure 1:**
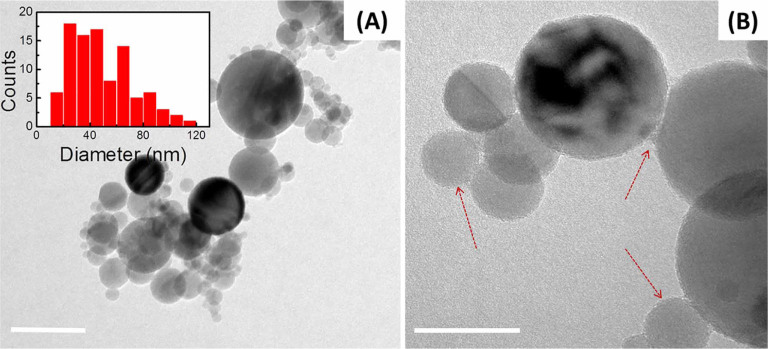
(A) BF-TEM *ex situ* images of as received alumina nanospheres with the diameter of the particle of measured as of 50 ± 26 nm (inset) (n = 132). Here the aggregation of nanoparticles is likely induced by the solvent evaporation during the TEM sample preparation. Inset shows particles size distribution. Scale bar: 50 nm. (B) A thin deposit on a surface of neighboring nanoparticles can be seen at higher magnification. Scale bar: 20 nm.

As-received powders were characterized by means of Bright Field TEM (BF-TEM). Figure 1 (A, B) shows the BF-TEM images of as received Al_2_O_3_ nanoparticles on a standard EM grid, with the mean diameter of the particle of measured as 50 ± 26 nm (n = 132), in good agreement with the equivalent spherical diameter calculated from the measured surface area of 38.8 m^2^/g^24^. Significant fraction (25−40%) of nanoparticles was found to exhibit a surface-localized deposit shown in Fig. 1(B).

**Figure 2:**
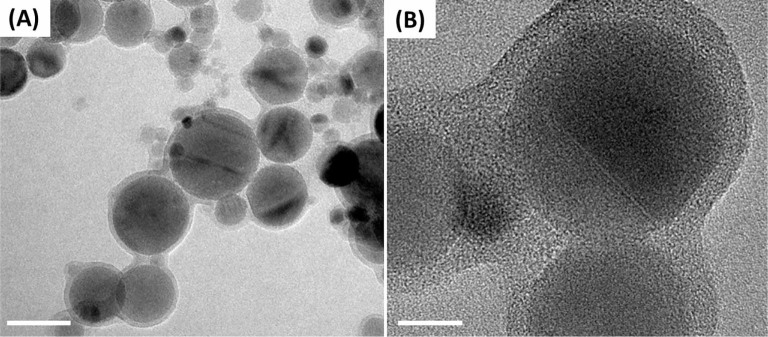
Disordered overlayer formation on neighboring nanoparticles. (A) BF-TEM image of the baked-in overlayer. Scale bar: 50 nm. (B) Higher-magnification image reveals amorphous nature of the formed overlayer. Scale bar: 10nm.

**Figure 3:**
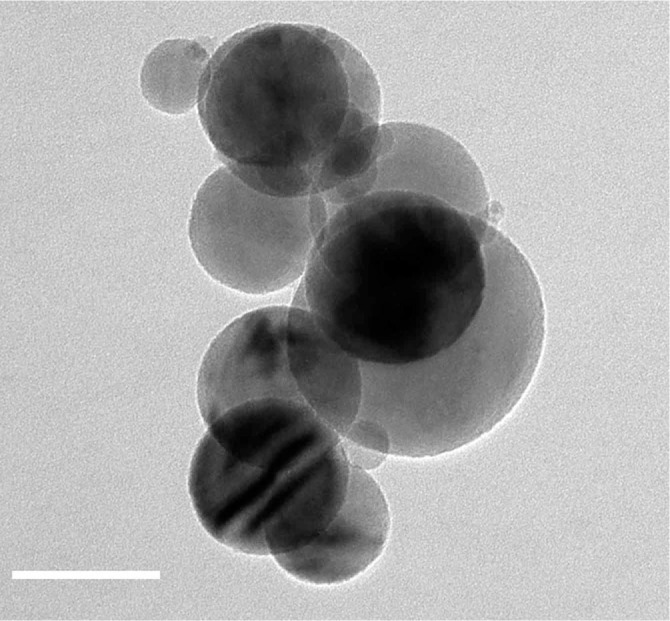
BF-TEM image of ethanol-modified alumina shows no trace of the previously observed surface deposit. Scale bar: 50 nm.

Figure 2 shows the disordered overlayer formed on a surface of neighboring nanoparticles of hydrated alumina powder dried at 125°C overnight. In Fig. 2 (A), individual nanoparticles appear to be linked together by such an overlayer. Figure 2 (B) shows a higher magnification image taken from a random sample area, with the amorphous overlayer of roughly 4 nm thickness encapsulating several crystalline particles at once. We attempted resetting the specimen history through the modification of the nanoparticles, described in experimental section. The result of the surface modification of the alumina nanopowder with ethanol, leading to removal of the overlayer formed on the powder surfaces, can be seen in the BF-TEM image in Fig. 3. Here the particle aggregation was attributed to the evaporation of solvent. Additional surface characterization was carried out by using the X-ray photoelectron Spectroscopy (XPS). Supp. Figure S1 shows the XPS spectra acquired on alumina powder exposed to different conditions.

**Figure 4:**
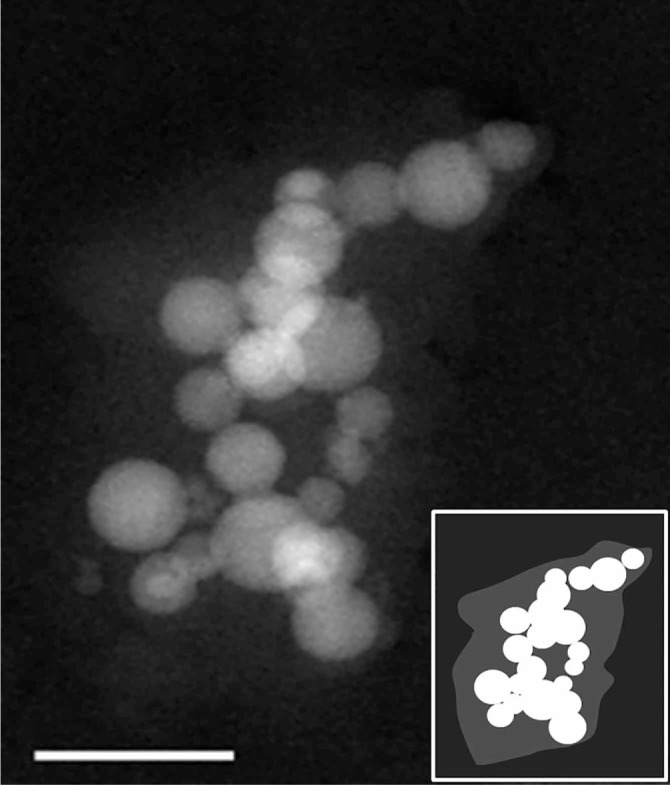
Cryo-HAADF-STEM image of diluted aqueous alumina slurry. The hydration layer is manifested as a faint cloud covering the aggregated nanoparticles. Scale bar: 100 nm. Inset shows the schematics of the hydrated aggregate.

Figure 4 shows the cryo-HAADF-STEM image of the hydrated alumina sample. The hydration layer formed around the aggregated particles, manifested as a lighter shadow, is schematically shown in the inset.

**Figure 5:**
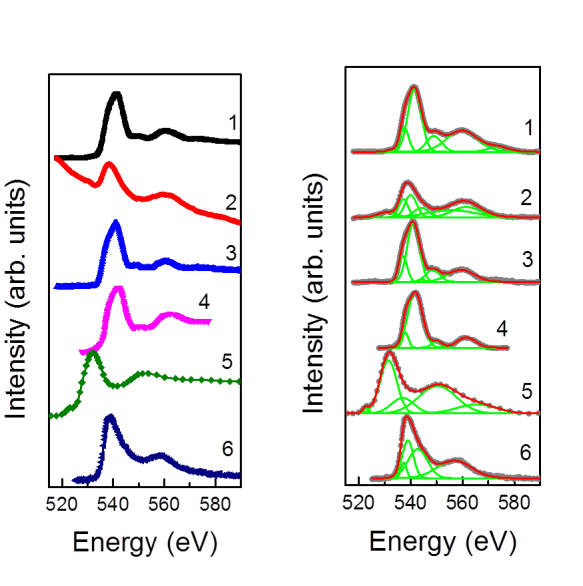
(A) *In situ* Electron Energy Loss O K-edge spectra acquired from (1), hydrated alumina, (2) ethanol-modified alumina, and the reference materials reported in the literature: (3) γ-Al_2_O_3_^29^, (4) α-Al(OH)_3_^30^ and (6) AlO(OH)^31^. (B) Peak fitting of the acquired spectra.

Figure 5 shows the EEL spectra of O K-edge peak acquired on specimens at different conditions. Figure 5 (A) presents the spectra from as-received alumina (**1**), hydrated alumina (**2**), ethanol-modified alumina (**3**), reference γ-Al_2_O_3_^29^ **(4)**, reference α-Al(OH)_3_^30^ **(5)** and reference AlO(OH)^31^ **(6)**. Figure 5 (B) shows peak fitting analysis of the acquired data and details of peak fitting are given in Table SI 1.

**Figure 6:**
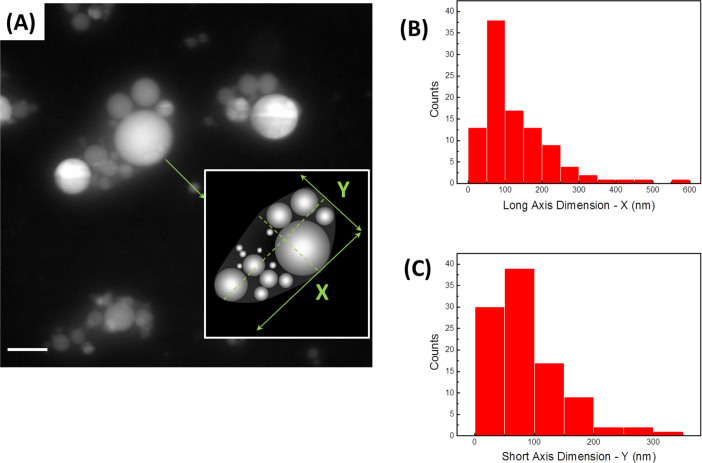
(A) *In situ* fluid cell HAADF STEM image of diluted aqueous alumina slurry. The hydration layer is manifested as a cloud enveloping aggregated nanopsheres. These hydrated aggregates and surrounding liquid represent the new nanoparticles in the slurries. Scale bar: 100 nm. Inset shows schematics of the formed aggregate with the size and aspect ratio different from that of initial spherical particles. (B) Size distribution of long axis, X, of the hydrated aggregates measured 133 ± 100 nm (n = 100). (C) Size distribution of short axis of the hydrated aggregates, Y, measured 87 ± 61 nm (n = 100).

Figure 6 (A) reveals the alumina nanoparticles in water, with the hydration layer manifested as a cloud enveloping several nanopsheres at once, as schematically shown in the inset. The aggregate is considered an ellipsoid with the mean values of the long and short axes of the aggregates formed in the liquid were reported in Fig. 6 (B, C) as 133 ± 100 nm and 87 ± 61.2 nm (n = 100), respectively. Here a relatively low image contrast had likely led to a larger error in the size measurements, contributing to a wider size distribution. Liquid cell holder allowed for limited tilting (± 22 degrees), and we have employed imaging of the tilted cell specifically to evaluate the third (thickness) dimension of the specimen. Based on the data obtained in this manner, the aggregation in Z-direction could be viewed as very similar to that in the direction of short (Y) axis.

## Discussion

Formation of aggregates observed in the dried sample (Fig. 1 (A)) could be attributed to an artifact created by capillary drying forces during the specimen preparation, which emphasizes the need to perform *in situ* imaging of the alumina nanoparticle suspensions to characterize their behavior in liquid. Material’s history represents one of important aspects working with nanostructured materials. While oxide nanoparticles are deemed stable, storing these materials under ambient conditions results in adsorption of atmospheric gases on the nanoparticles’ surface and leads to formation of a thin deposit shown in Fig. 1 (B). Such a surface-bound buildup, apparently formed over several nanoparticles, is likely to affect the properties of the nanopowder upon the contact with water, specifically the surface-localized hydration. In Fig. 2, the presence of disordered overlayer formed over the surface of nanoparticles, can be attributed to the elusive hydration layer stabilized *via* the baking of specimen, as shown in Fig. 2 (A). A notable aggregation of alumina nanoparticles presented in Fig. 2 (A), can be explained by formation of a disordered surface-bound overlayer over the neighboring nanoparticles forming clusters, as exemplified in Fig. 2 (B). Such a process is consistent with a noted decrease in a surface area of the hydrated powder upon drying, from 38.8 m^2^/g to roughly 15 m^2^/g according to Brunauer–Emmett–Teller (BET) surface area measurements. Use of ethanol modification allowed complete removal of the surface deposit (Fig. 3). Surface modification, therefore, could be used as an effective means to clean the nanoparticle surfaces.

Figure 4 shows the HAADF-STEM image of the vitrified hydrated alumina. Here aggregation of nanoparticles likely reflects the overall interparticle interactions in the liquid state. This solution-aggregated state is preserved during the cryo-plunging procedure, where the vitrification takes place almost instantaneously and does not allow the aggregate to disassemble in the process. Upon close examination of the cryogenic image, the faint layer enveloping the aggregate becomes apparent. It is worth noting that the thin amorphous carbon substrate grid adds to the overall image intensity and contributes to the low contrast in the cryogenic image. Additional factors affecting the image contrast are related to the overall thickness of the specimen and the cryo-EM imaging requirement to maintain the so-called *low dose*. This results in a lower number of electrons reaching the CCD detector and contributing to the overall noise, therefore adversely affecting the image contrast^32^,^33^.

The local chemical environment of aluminum was probed *in situ* by EELS using the reference spectra for O K edge, as shown in Fig. 5 (A). The qualitative changes in the chemistry of alumina nanoparticles and the surrounding disordered hydration layer were evaluated by monitoring the evolution of this peak. Several types of surface complexation models have been reported by various groups in an attempt to adequately describe the surface properties of alumina nanoparticles and their interaction with the solvent^29^,^34^,^35^,^36^,^37^,^38^,^39^,^40^,^41^,^42^,^43^,^44^. It is worth noting, however, that the majority of reports pertained to the data acquired *ex situ* by employing the combination of thermal analysis (DTA, TGA) and conventional spectroscopic techniques, such as infrared spectroscopy (IR) and XPS. Notably, Lefèrve and co-authors reported on hydroxylation of γ-Al_2_O_3_ and formation of Al(OH)_3_ and AlO(OH) upon the exposure to water^37^. The spectra acquired on as-received alumina (spectrum 1), hydrated alumina (spectrum 2) and ethanol-modified alumina (spectrum 3) are compared to those of reference materials reported in the literature, namely γ-Al_2_O_3 _(spectrum 4)^29^, Al(OH)_3_ (spectrum 5)^30^ and AlO(OH) (spectrum 6)^31^, respectively. The peak fitting analysis presented in Fig. 5 (B) indicates the presence of several compounds formed as a result of the hydration process. The EELS results are listed in Supp. Table SI 1. The peaks around 541, 549 and 560 eV in as-received alumina (spectrum 1), hydrated alumina (spectrum 2) and ethanol-modified alumina (spectrum 3) match the reported characteristic peaks for γ-Al_2_O_3_^29^. The hydroxylation of the as-received (spectrum1) and hydrated samples (spectrum2) is evidenced by the presence of peaks around 531 eV, which is, reportedly, one of the fingerprints for Al(OH)_3_^30^. The absence of this hydroxylated peak in ethanol-modified sample confirms the effective removal of the initial hydration layer *via* ethanol treatment. The presence of the peak at 557 eV in hydrated sample (spectrum 2) also suggests the presence of AlO(OH)^31^.

In current study, presence of the hydration layer in aqueous alumina colloidal solution was directly visualized in liquid using the STEM *in situ* holder platform (cf. Supp. Figure S2). It was initially assumed that once the powder is exposed to water, the hydration layer would only form around the individual nanoparticles. However, the majority of the nanoparticles were found to aggregate, with the hydration layer forming around the aggregates. Figure 6 (A) reveals the alumina nanoparticles in water, with the hydration layer manifested as a cloud enveloping several nanopsheres at once, as schematically shown in the inset of Fig. 6 (A). The inner region of the aggregate exhibits a relatively constant Z contrast throughout, consistent with a relatively constant concentration of aluminum atoms. The hydration layer cloud, on the other hand, showing weaker contrast, diminishes at about 64 nm from the aggregate boundary. This is indicative of depletion of aluminum ions in the solution surrounding the aggregate. From image analysis, the thickness of this hydration layer was estimated to be approximately 64 ± 17 nm (n = 31).

Upon exposure to water, the surface of nanoparticles likely undergoes a rapid hydration and contributes to formation of the solvated cloud enshrouding the entire aggregate. The hydrated aggregates represent the *new type of particles*, with much larger size and high aspect ratio. Therefore, rheological behavior of such a system would, most certainly, deviate from the theoretical predictions. To account for the effect of apparent aggregation of the particles in liquid and formation of the hydration layer over the aggregates on the viscosity of alumina suspension, the aggregates are viewed as new particles with the dimensions drawn along the two perpendicular axes, as schematically shown in the inset of Fig. 6 (A). The mean values of these long and short axes of the aggregates formed in the liquid are measured as 133 ± 100 nm and 87 ± 61 nm (n = 100), respectively, as shown in Figs. 6 (B, C). A relatively low image contrast had likely led to an error in size measurements, contributing to a broader size distribution.

Suspensions of alumina nanoparticles exhibit higher viscosity compared to the predicted values^45^,^46^. Using the mean diameter of individual nanoparticles, the viscosity of the as-received colloidal alumina suspension was calculated using the Krieger-Dougherty equation^28^,
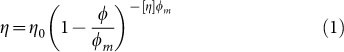
where *η* is the viscosity of the suspension, *η_0_*is the viscosity of solvent (0.89 mPa·s for water at 25^o^C), *ϕ* is the solids content, *ϕ_m_* is the maximum packing of nanoparticles in the colloidal suspension (*ϕ_m_* = 0.64 for random packing of monodispersed spherical particles) and [*η*] is the intrinsic viscosity (*[η]* = 2.5 1. for spherical nanoparticles)^47^. According to the Krieger−Dougherty relation, the viscosity of alumina suspensions with solids content of 0.05 was computed as 1.0 mPa·s, lower than the experimental viscosity values reported by Çınar and co-authors (*η* = 2.0 ± 0.4 mPa·s at the shear rate of 10 s^–1^^48^). The intrinsic viscosity [*η*] in the Krieger-Dougherty equation is now defined as a function of the long (X) and short (Y = Z) axes of the aggregates^49^. The higher-magnification images were used to evaluate the effect of the formed aggregates on the intrinsic viscosity with higher precision (Fig. 6A). The long (X) and short (Y) axes were measured as 180 nm ± 104 nm and 108 ± 56 nm for these 17 hydrated aggregates, respectively, while the thickness of the aggregates (Z) was assumed equal to that of short (Y) axis. The intrinsic viscosity for this aspect ratio would correspond to [η] = 2.90^49^. In addition to the intrinsic viscosity change, the hydration layer, formed over the aggregate, is now a part of a particle in the suspension, increasing the effective solids content of the suspension by 240 ± 150 % due to the formation of hydration layer over the aggregates. Using [η] = 2.90, *ϕ* = 0.18, and *ϕ_m_* = 0.91 (including the volume contribution of the hydrated layer estimated from the DSC experiments^48^), the viscosity of alumina suspensions was calculated as 1.6 mPa·s for the solids content of 0.05. Accounting for the formation of hydrated aggregates, the calculated viscosity values for alumina suspensions with the 0.05 solids content now fall into the experimental viscosity range reported by Çınar and co-workers^48^. Relatively lower predictions of the suspension viscosity were expected as the effect of interparticle interactions were excluded in the model, which is particularly significant for the highly loaded systems of nanoparticles. Incorporating the updated intrinsic viscosity and accounting for the drastically increased solid content, the Krieger-Dougherty equation, therefore, provides realistic viscosity values.

## Conclusions

The presence of the hydration layer in alumina suspension was first established *ex situ* using the hydrated alumina powder dried at 125°C overnight representing a 4 nm disordered overlayer on the surface of the nanoparticles. We probed the nature of this overlayer and identified the protocol suitable for effective modification of the alumina particles surface.

We have described a new approach for visualization of oxide surfaces in aqueous systems, thus providing critical information for many technologically significant applications. Hydration layer formation around alumina aggregates was detected in vitrified samples *via* cryo-HAADF-STEM imaging. Next, using *in situ* fluid cell STEM holder platform, we have directly visualized, for the first time, the presence of hydrated layer in aqueous alumina suspensions in liquid. We show that in aqueous solutions alumina nanoparticles aggregate to form hydrated clusters of significantly larger sizes with high aspect ratio. Formation of hydrated aggregates drastically increases the effective solids content and decreases available free liquid carrier of the suspensions, resulting in high viscosities. Our findings explain the discrepancy between the theoretical viscosities calculated for systems comprised of monodisperse nanoparticles and the viscosity values experimentally determined in actual colloidal suspensions, where the nanoparticles exist in form of hydrated aggregates. By employing the hydrated aggregate dimensions in the Krieger-Dougherty equation we were able to reconcile the disparity between the model predictions and the experimentally determined viscosities and hence demystified the hydration layer formed on alumina nanoparticles in aqueous suspensions. Our findings prompt the revision of the parameters employed in forecasting of viscosity of highly concentrated nanoparticle colloidal suspensions and necessitate the establishment of new realistic models based on the observed hydration behavior of nanoparticles in aqueous suspensions.

## Methods

### Materials and Reagents

Alumina nanoparticles (99.99%, Nanophase Technology Corporation, Burr Ridge, IL, USA, Lot numbers: AAGL 1201, AAGL 1203) and ethanol (Decon Labs, USA, 200 Proof) were used as-received. All aqueous solutions were prepared with deionized water passed through a Millipore Milli-Q Plus water purification system (λ = 18.2 MΩ-cm). Surface modification of alumina powder was carried out as follows. Aqueous alumina suspensions were subjected to centrifugation at 6000 RPM for 10 minutes, and removing the supernatant. The precipitate was then dehydrated by being transferred into ethanol, vigorously shaken and left in ethanol over 72 h, followed by the centrifugation at 6000 RPM for 10 minutes (x3) and removal of the supernatant, after which the modified powder was re-suspended in ethanol and used for analysis.

### Conventional Room Temperature EM Characterization *ex situ*

2 μL droplet of diluted alumina suspension was deposited on conventional carbon-coated copper and gold grids (QuantiFoil™), allowed to dry, and subjected to BF-TEM and HAADF-STEM imaging using a standard single-tilt holder.

### Cryo-EM Characterization

The 300 mesh carbon-coated copper grids (QuantiFoil™) were used in this work. The grids were glow-discharged (Pelco easiGlow, Ted Pella, USA) for 20 seconds. 3 μL of diluted alumina suspensions were deposited on the grid, blotted, for 1–2 seconds, cryo-plunged (Cryoplunge, Gatan, USA) into liquified ethane (99.95%, Matheson Gas, USA), and stored in liquid nitrogen. The grids were loaded in the cryo-workstation to the 626 single tilt liquid nitrogen cryotransfer holder (Gatan, USA), transferred to the Tecnai G^2^ F20 STEM, and imaged in BF-TEM and HAADF STEM modes.

### Fluid Cell Characterization *In situ*

The fluid cell STEM imaging is schematically shown in Figure SI 2. The alumina nanoparticle suspensions were examined with a Continuous Flow Fluid Cell TEM Holder Platform (Hummingbird Scientific). Silicon nitride window membranes were cleaned by rinsing in toluene (3 × 3 mL), rinsed with chemically pure acetone (3 × 3 mL) and washed with ethanol (3 × 5 mL), followed by cleaning in ozone plasma cleaner (BioForce) for 45 minutes. The alumina suspensions were deposited onto plasma-cleaned electron-transparent silicon nitride window membranes for *in situ* imaging. After the specimen deposition, the windows were assembled and sealed, resulting in the liquid specimen sandwiched between the electron-transparent silicon nitride window membranes. *In situ* fluid delivery was carried out with a syringe pump with the variable pumping speed (2–5 µL/min).

Imaging and characterization of the specimens was carried out with an FEI Tecnai G^2^ F20 (S)TEM operating at an accelerating voltage of 200 kV equipped with a Tridiem Gatan Imaging Filter (GIF), High Angle Annular Dark Field (HAADF) and Energy Dispersive X-ray Spectroscopy (EDS) detectors. Electron Energy Loss Spectroscopy (EELS) was used to probe the localized chemical composition of the specimens. Data and image analyses were performed by using Digital Micrograph (GMS version 2.11.1404.0), ES Vision 5.0, ZEN 2012 and OriginPro 9.0 software. To ensure reproducibility of results, mean particle size (n = 132) and long and short axes measurements of random aggregates (n = 100) were analyzed in the numerous micrographs obtained in the BF-TEM and HAADF STEM modes, respectively.

### X-ray Photoelectron Spectroscopy

Alumina nanopowders were pressed into indium foil in the inert dry atmosphere glove box and inserted into the FPI5500 X-Ray Photoelectron spectrometer using the transfer chamber, where the specimen was allowed to equilibrate at the base pressure of 1.2 × 10^–10^ Torr. Al *K* line at 1486.6 eV (250 W), with the emission angle 45 degrees, was used with the pass energy 187.85 eV for the surveys and 58.7 eV for the multiplex scans. Detailed data acquisition in the regions of interest was performed on three separate areas of the specimen for an extended period of time (10 min, 22 acquisitions). Argon plasma etching was carried out in-situ for 10 min at the pressure levels not exceeding 8.5 × 10^–8^ Torr, after which the data acquisition (22 acquisitions) was repeated. The depth profiling in this study was evaluated by using that of SiO_2_/Si^50^. Specimen charging was measured by the displacement of the adventitious carbon C1*s* line from 284.8 eV and ranged from 0.1 to 6.1 eV. Corrections to the binding energy values for the samples were made using the carbon charging shift values.

## Author contributions

SK and TP performed fluid cell STEM experiments; SK and EF carried out EFTEM imaging ex-situ; EF performed cryo-TEM characterization and data analysis. SC conceived the idea and participated in experiments and discussion of results. EF and TP drafted the manuscript. The manuscript was written through contributions of all authors.

## Additional information

**Supplementary information** accompanies this paper at http://www.nature.com/scientificreports

**How to cite this article:** Firlar, E., Çinar, S., Kashyap, S., Akinc, M. & Prozorov, T. Direct Visualization of the Hydration Layer on Alumina Nanoparticles with the Fluid Cell STEM *in situ. Sci. Rep.* 5, 9830; DOI:10.1038/srep09830 (2015).

## Supplementary Material

Supplementary Information Supplementary Information
