# Supplementary material for: Direct Visualization of the Hydration Layer on Alumina Nanoparticles with the Fluid Cell STEM in situ
Source: Sci Rep. 2015 May 21;5:9830. doi: 10.1038/srep09830 (PMC4440531; doi:10.1038/srep09830)
Supplement: Supplementary Information [file srep09830-s1.pdf]

Supplementary Material

Direct Visualization of the Hydration Layer on Alumina Nanoparticles with the Fluid Cell STEM in situ

Emre Firlar[†], *Simge Çinar*[‡]*, *Sanjay Kashyap*[†*], *Mufit Akinc*[†‡§] *and Tanya Prozorov*[†§]

[†]Division of Materials Science and Engineering, US DOE Ames Laboratory, Ames IA, 50011, USA
[‡]Department of Materials Science and Engineering, Iowa State University, Ames IA, 50011, USA

* These authors contributed equally to the project

[§] Address correspondence to

Phone: +1-515-294-0738. Fax: +1-515-294-5444. E-mail: makinc@iastate.edu

Phone: +1-515-294-3376. Fax: +1-515-294-8727. E-mail: tprozoro@ameslab.gov

XPS analysis was used to monitor the surface evolution of alumina powders exposed to different conditions, as shown in **Figure S1**. Using the best-fit peak analysis, several spectral signatures present in the XPS peaks could be, tentatively, assigned to various aluminum oxides and hydroxides. As-received powder exhibits surface deposit attributed to the absorbed atmospheric gases, associated with the lower-energy carbon peak. Examination of O 1s spectra shows that exposed to atmosphere powder exhibits some degree of hydroxylation, as evidenced by the interplay of the oxidic and hydroxidic peaks reported by Lefèrve and co-authors [37]. Drying the hydrated powder at 40 ^{o}C overnight reduces the amount of the absorbed gas, decreases the hydroxidic component, and leads to the overall homogenization of the surface. Ethanol-modified powder exhibits a single-peak oxygen spectrum, providing further evidence of the effective removal of surface deposit observed by the BF TEM.

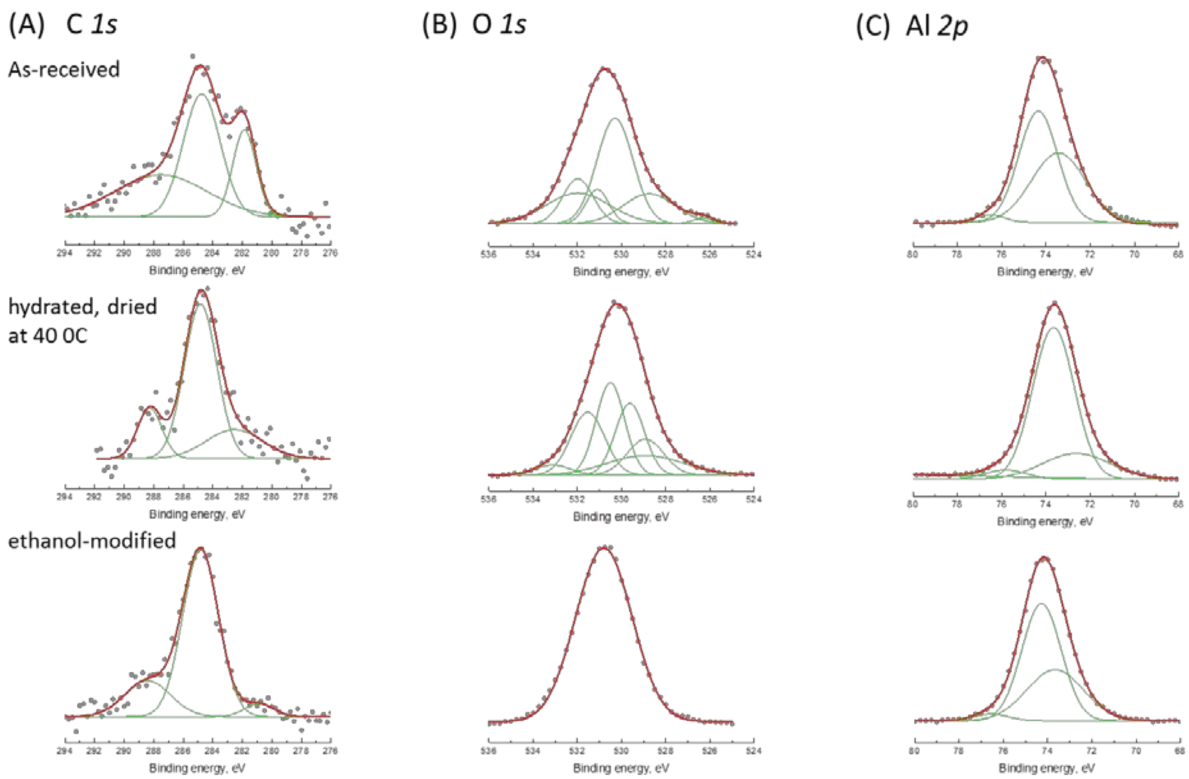

Figure S1 XPS spectra acquired on alumina powder exposed to different conditions: (A) C*1s* peaks, (B) O*1s* peaks, (C) Al*2p* peaks.

Table S1 Peak fitting analysis of the EELS spectra presented in **Figure 5.**

	Peak Index	Center Max (eV)	Max Height	FWHM (eV)
1 (as-received alumina)	1	532.54	0.03	4.19
	2	537.57	0.37	3.88
	3	541.52	0.96	6.08
	4	548.98	0.24	6.86
	5	559.82	0.33	13.97

	6	573.92	0.07	9.7
2 (hydrated alumina)	1	530.98	0.08	8.12
	2	537.22	0.28	4.24
	3	540.04	0.34	5.32
	4	544.16	0.14	6.7
	5	550.34	0.02	1.22
	6	555.73	0.11	21.88
	7	561.49	0.16	12.75
3 (ethanol-modified alumina nanoparticles)	1	537.08	0.39	3.77
	2	541.05	0.9	5.53
	3	544.5	0.04	2.47
	4	548.44	0.18	6.43
	5	559.45	0.18	12.1
4 (γ-Al_2O_3 from Reference [29])	1	538.02	0.23	2.74
	2	541.73	0.85	6.57
	3	550.19	0.1	4.58
	4	561.54	0.16	8.4
5 ($Al(OH)_3$) from Reference [30])	1	522.64	0.09	2.79
	2	531.42	0.81	7.65

	3	536.95	0.24	10.26
	4	550.36	0.42	16.95
	5	565.46	0.14	18.05
6 (AlO(OH) from Reference [31])	1	537.34	0.24	2.52
	2	538.92	0.58	4.97
	3	542.71	0.44	8.84
	4	556.87	0.27	15.73

Using the peak fitting analyses, the EEL spectra obtained for O K-edge were analyzed and tried to be related to the O K-edges of reference γ-Al_2O_3 [29] **(4)**, reference α- $Al(OH)_3$ [30] **(5)** and reference AlO(OH) [31] (6).

Using the fluid cell STEM imaging schematically shown in **Figure SI 2,** it was possible to visualize alumina nanoparticle suspensions in liquid, their fully hydrated state *in situ*. It is worth noting, that such an experiment cannot be carried out using any conventional microscopy technique. In the current work, alumina nanoparticle suspension was sandwiched between the Si_3N_4 windows and imaged with a focused scanning STEM probe in the thin liquid layer.

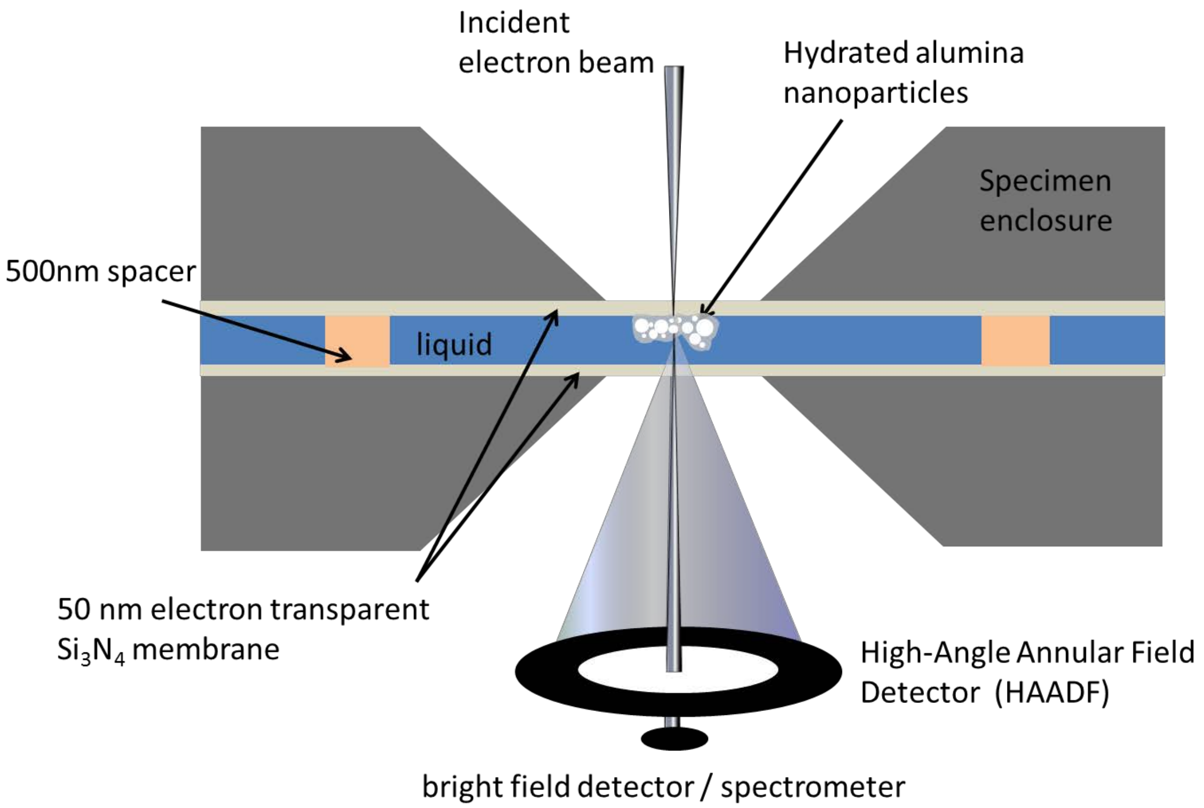

Figure S2 In situ fluid cell STEM schematics. Alumina nanoparticle suspensions are sandwiched between the two electron-transparent Si_3N_4 windows and imaged with a focused scanning STEM probe in the thin liquid layer.
